# Integration of exteroceptive and interoceptive information within the hippocampus: a computational study

**DOI:** 10.3389/fnsys.2015.00087

**Published:** 2015-06-05

**Authors:** Randa Kassab, Frédéric Alexandre

**Affiliations:** ^1^INRIA Bordeaux Sud-OuestTalence, France; ^2^LaBRI, UMR 5800, Centre National de la Recherche Scientifique, Bordeaux INP, Université de BordeauxTalence, France; ^3^Institut des Maladies Neurodégénératives, UMR 5293, Centre National de la Recherche Scientifique, Université de BordeauxBordeaux, France

**Keywords:** episodic memory, single-trial learning, binding, valence, interference, hippocampal-dependent behaviors

## Abstract

Many episodic memory studies have critically implicated the hippocampus in the rapid binding of sensory information from the perception of the external environment, reported by exteroception. Other structures in the medial temporal lobe, especially the amygdala, have been more specifically linked with emotional dimension of episodic memories, reported by interoception. The hippocampal projection to the amygdala is proposed as a substrate important for the formation of extero-interoceptive associations, allowing adaptive behaviors based on past experiences. Recently growing evidence suggests that hippocampal activity observed in a wide range of behavioral tasks could reflect associations between exteroceptive patterns and their emotional valences. The hippocampal computational models, therefore, need to be updated to elaborate better interpretation of hippocampal-dependent behaviors. In earlier models, interoceptive features, if not neglected, are bound together with other exteroceptive features through autoassociative learning mechanisms. This way of binding integrates both kinds of features at the same level, which is not always suitable for example in the case of pattern completion. Based on the anatomical and functional heterogeneity along the septotemporal and transverse axes of the hippocampus, we suggest instead that distinct hippocampal subregions may be engaged in the representation of these different types of information, each stored apart in autoassociative memories but linked together in a heteroassociative way. The model is developed within the hard constraint of rapid, even single trial, learning of episodic memories. The performance of the model is assessed quantitatively and its resistance to interference is demonstrated through a series of numerical experiments. An experiment of reversal learning in patients with amnesic cognitive impairment is also reproduced.

## 1. Introduction

Since Tulving's (1972) proposal to consider the concept of episodic memory as a specific form of declarative memory that allows us to explicitly remember individually experienced events within their context, a consensus has been emerging that the hippocampus, a medial temporal lobe structure, is crucially involved in the encoding, storage and retrieval of spatial and nonspatial episodic memories (Cohen and Eichenbaum, 1993; Eichenbaum et al., 2012; Rolls, 2013). Equally, a number of emotional and instrumental behaviors show a similar dependence on the hippocampus (Kennedy and Shapiro, 2009; Shohamy et al., 2009; Eichenbaum et al., 2012; Maren, 2014). Memory of the context in which a reward or punishment has been received can become reactivated through explicit or implicit recall processes (Lisman and Redish, 2009). This reactivation is thought of as a prospective use of episodic memory traces for anticipating future events and selecting strategic actions (Lee et al., 2006; Shohamy et al., 2009).

In both cases, the specific contribution of the hippocampus is related to its unique ability to rapidly learn and use bindings of arbitrary relations among separate perceptual features of an experience (Cohen and Eichenbaum, 1993; O'Reilly and Rudy, 2001). This binding has often been considered to be flexible in the sense that established memories can be reactivated by related retrieval cues in novel situations that are different from the original situation in which learning took place. It has been suggested that all hippocampal subregions [dentate gyrus (DG), CA3, and CA1] are important for the processing of episodic memory, with each subregion mediating distinct but interdependent computations, e.g., pattern separation in the DG (Leutgeb et al., 2007; Rolls, 2013), pattern completion in CA3 (Rolls, 2013), matching and novelty detection in CA1 (Hasselmo et al., 1996). Processing of spatial and non-spatial information, however, reveals the existence of a functional segregation along the transverse hippocampal axis in CA1 (Tamamaki and Nojyo, 1995), and, more recently, in CA3 as well (Nakamura et al., 2013). Recent studies have also drawn attention to the fact that CA3 is not a uniform network with completely random connections (de Almeida et al., 2007; Witter, 2007; Bush et al., 2010; Nolan et al., 2011; Kesner, 2013). Three subregions, CA3a, b, and c, are usually identified. Both CA3a and CA3b (close to CA1) have strong recurrent connections and relatively few connections with CA1, whereas CA3c (close to the DG) has relatively few recurrent connections but sends strong projections to CA1 (Hunsaker et al., 2008).

Strong evidence for functional differentiation along the septotemporal axis has also been derived from anatomical, behavioral, and gene expression studies (Moser and Moser, 1998; Thompson et al., 2008; Fanselow and Dong, 2010). The dorsal/septal and ventral/temporal poles of the hippocampus differ strikingly in their afferent and efferent connections to cortical and subcortical structures. The dorsal hippocampus has—via the entorhinal cortex (EC)—bidirectional connectivity with various cortical regions known to be implicated in the processing of visuospatial information, whereas the ventral hippocampus is more strongly connected to regions implicated in emotional and motivational behaviors, such as hypothalamus, prefrontal cortex (PFC), amygdala and insular cortex (van Groen and Wyss, 1990; Pitkänen et al., 2000; Cenquizca and Swanson, 2007). Consistent with this pattern of connectivity, dorsal hippocampal lesions have been found to disrupt spatial memory whereas ventral lesions alter stress and emotional functions (Henke, 1990; Moser et al., 1995; Kjelstrup et al., 2002). At the moment evidence grows in support of a more refined division of the septotemporal axis of the hippocampus (Thompson et al., 2008; Bast et al., 2009; Strange et al., 2014).

The hippocampus and its associated functions have been extensively studied in modeling works. To date, however, it has been proved difficult to render hippocampal models compatible with both episodic memory and other cognitive functions that involve volitional and reflex motor responses (e.g., classical conditioning Gluck et al., 2003). Models that underlie episodic memory should be able to rapidly encode neural representations of single experiences, but models that seek to explain hippocampal involvement in emotional and behavioral tasks require many training trials for learning to be effective (O'Reilly and Rudy, 2001). These later roughly fall into one of two categories as described below.

In the first category are models based on the assumption that the role of the hippocampus is restricted to the association or binding of sensory features that depend on the perception of the external environment (exteroception; see Table 1). They take the view that the formation of extero-interoceptive associations lies exclusively in the change in the synaptic strength of extrinsic connections between the hippocampus and other emotion-related structures, like the amygdala (LeDoux, 2007), especially its basolateral nucleus (BLA) (Eichenbaum et al., 2012), relying on error-driven learning mechanisms (Schmajuk and DiCarlo, 1992; Meeter et al., 2005). This kind of learning appears to be consistent with the slow acquisition of various conditioned behaviors, but does not seem to delineate a complete picture of the functional role of the hippocampus. A number of context-dependent behaviors change as the result of a single, past experience. For example, (Wiltgen et al., 2006) demonstrated that contextual fear learning can be acquired after a single conditioning trial when the hippocampus is intact. Rapid behavioral reversal in early extinction trials is likely to be driven by the hippocampus (Shohamy et al., 2009). It is suggested by the same token that the hippocampus may be involved in the immediate recovery of fear responses as observed after extinction in the amygdala (Hobin et al., 2006). These data, together with the high connectivity of the hippocampus with interoceptive perceptual systems (Pitkänen et al., 2000; Craig, 2009), raise the possibility to extend the rapid binding in the hippocampus to capture interoceptive features of episodic memories.

**Table 1:** **Glossary**.

**Term**	**Description**
Exteroception	The perception of environmental stimuli originating outside of the body, e.g., visual, acoustic, or tactile stimuli.
Interoception	The perception of the body's internal state through the processing of signals arising from within the body, e.g., blood pressure, heart beats, etc. Interoceptive features may reflect the emotional valence, arousal and other somatic states.
Valence	One of the most commonly described dimensions of emotions that ranges from highly positive to highly negative according to how pleasant or unpleasant a stimulus might be.
Arousal	The activation dimension that ranges from calm to excitement.
Valence-overload	A condition that occurs when a stimulus or a cue is simultaneously associated to different, sometimes contradictory, valences.
Valence-overload interference	A decrement in the ability of a memory system to reliably remember previously formed associations between exteroceptive stimuli and their emotional valences.

Now, it is known from neurophysiological data that hippocampal activity is related to the valence (e.g., reward, neutral, or punishment) of experiences. There is evidence that CA3 contains a representation of reward-place associations that may be used to remember goals available at different locations (Rolls and Xiang, 2005). Selective neuronal responses to reward delivery have been recorded in the hippocampus (Smith and Mizumori, 2006) and in the EC (Sugase-Miyamoto and Richmond, 2007). Furthermore, activity in CA1 has been observed to signal learning-related prediction errors in reference to past experiences (Wirth et al., 2009; Lee et al., 2012).

The second category of models is coherent with these findings, being developed with a consideration of interoceptive features, specifically emotional valences, as a relevant part of episodic memories (Gluck and Myers, 1993; O'Reilly and Rudy, 2001; Moustafa et al., 2009; Rolls, 2010). A “flat” representation which concatenates all the perceived sensory features, both exteroceptive and interoceptive, is used and stored in a single autoassociative network which supports pattern completion. These models have proved to be effective for valence prediction and to account for a wide range of classical conditioning tasks. However, as others before, neural representations are gradually shaped over a significant number of training trials, a compelling reason why they are thought to be incompatible with the widely held view of single-trial hippocampal acquisition of episodic memories and other forms of one-experience context-dependent tasks (Gluck et al., 2003). Yet, all of these models, mostly oriented toward hippocampal involvement in adaptive behaviors, have not attempted to explicitly ascertain their performance under different recall conditions. The quality of valence prediction was tested using all of the previously encountered exteroceptive cues associated with learning. However, recall is usually believed to occur when subsets of exteroceptive cues of past experiences are reactivated. Simply binding extero-interoceptive features onto a single pattern of activity as proposed above implies that, under recall conditions with partial exteroceptive cues, a pattern completion process would concurrently fill in what is missing from exteroceptive and interoceptive patterns. This may result in impaired performance on valence prediction relative to that under full-cue recall conditions.

In sum, at the biological level of analysis, a great deal of evidence exists to support the view that extero-interoceptive associations are learned both within and outside the hippocampus. So high plasticity in the hippocampus may represent the neural substrate for the rapid acquisition of some hippocampally-dependent behaviors, even in a single trial as may occur in episodic memory. Other more slowly acquired behaviors may depend on incremental learning involving other brain structures, like the amygdala, but remain nevertheless dependent on the hippocampus as a primary source of contextual inputs. On the other hand, to our knowledge, no computational models have been developed to explain how extero-interoceptive associations might be rapidly formed within the hippocampus. Indeed, single-trial learning is a hard learning problem, particularly when emotional memories have to be considered (Mather, 2009). Take, for example, the case of three emotionally valenced patterns AB+, AC−, and BD−. It is possible to store each of these patterns by strengthening associative links between its three constituent features. However, exteroceptive features, like A and B, which are shared among positively and negatively valenced patterns, will become paired with both positive and negative valences. This will, of course, lead to a wrong prediction of AB valence when presented as a retrieval cue to the network. This type of interference, which we will refer to as “valence overload,” can be solved by incremental learning through a series of presentations of the set of input patterns in order to gradually discover their latent structure and adjust neural representations of interfering patterns. The computational challenge that needs to be addressed, however, is how to go about doing so under the condition of rapid learning which may be the natural province of the hippocampus.

The computational model that we describe in this paper is the first to address the rapid, one-trial, binding of extero-interoceptive features within the hippocampus. In line with the anatomical and functional heterogeneity of the hippocampus, our model is based around an integrated network of interconnected auto- and heteroassociative memories. Instead of the purely autoassociative way of binding proposed by previous models, neural assemblies representing exteroceptive sensory inputs and their emotional valences are considered as two sets of independent features, each stored apart in autoassociative memories that are linked in a heteroassociative way. This implies a distinction between two specific forms of interference, namely “pattern overload” and “valence overload,” that can occur respectively at the level of the autoassociative and heteroassociative memories. Pattern overload has been extensively addressed under the assumption that the DG implements pattern separation mechanisms to ensure the successful encoding of distinct memory traces in the CA3 autoassociative network (Rolls, 2010). Therefore, we will focus primarily here on demonstrating the ability of the model to rapidly link exteroceptive patterns and their emotional valences while avoiding valence-overload interference.

## 2. Materials and methods

### 2.1. Network architecture and computational principles

**Figure 1:**
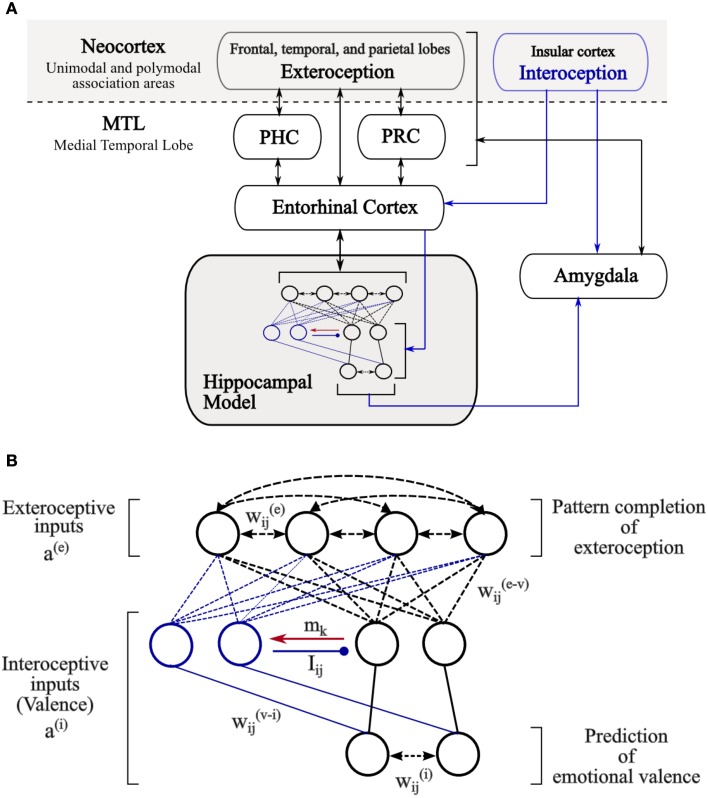
**The architecture of the hippocampal model**. **(A)** General schematic diagram showing the input-output relationships that the model is hypothesized to have with other brain regions. Black arrows indicate flow of exteroceptive information while blue arrows indicate flow of interoceptive information among the identified regions. Abbreviations: PHC, parahippocampal cortex; PRC, perirhinal cortex. **(B)** The hippocampal model implemented in this study. Network inputs correspond to the activities of cells in the entorhinal cortex, which respond to exteroceptive features as well as interoceptive, valence, states related to external stimuli. Black lines denote the basic circuit of the model while blue lines denote changes in circuitry mediated by valence-associated cells (blue) following the detection of valence-overload interference (red arrow). Autoassociative and heteroassociative connectivities between hippocampal cells are denoted respectively by bidirectional dashed lines and simple dashed lines without arrows. Inhibitory connections between valence cells are denoted by lines ended with circles. Stable non-plastic connections, both excitatory and inhibitory, are denoted by solid lines.

The major component of the proposed model is the hippocampal network (Figure 1). The input-output relations between the hippocampal network and other brain areas are implemented as distinct patterns of activity across populations of cells in the entorhinal cortex (EC). The computational implementation details can be found in Table 2.

**Table 2:** **Model equations for learning and recall processes**.

**#**	**Equation**	**Description**
**RECALL**		
R1	$\begin{array}{l} {\hat{a}}_{j}^{\left(e\right)}=H(\sum_{i=1}^{n}w_{ij}^{\left(e\right)}a_{i}^{\left(e\right)}-\sum_{i=1}^{n}a_{i}^{\left(e\right)})\\ H(x)=\{\begin{array}{l} 1,\;\;\;\;\;\text{if}\;\;x\geq 0\\ 0,\;\;\;\;\;\text{if}\;\;x<0 \end{array} \end{array}$	The exteroceptive autoassociative network is presented a retrieval cue, *a*^(*e*)^ ∈ {0, 1}^*n*^, from the entorhinal cortex (EC). The retrieval activity pattern $\hat{a}$^(*e*)^ ∈ {0, 1}^*n*^ is obtained by summing dendritic input from the recurrent connections for each cell, *j*, and applying a firing threshold $\theta =\sum_{i}^{n}{\tilde{a}}_{i}^{\left(e\right)}$.
R2	$\begin{array}{l} y_{l}^{\left(v\right)}={\hat{y}}_{l}^{\left(v\right)}\wedge {\bar{I}}_{l}\\ {\hat{y}}_{l}^{\left(v\right)}=H(\sum_{j=1}^{n}w_{jl}^{\left(e-v\right)}{\hat{a}}_{j}^{\left(e\right)}-\sum_{j}^{n}{\hat{a}}_{j}^{\left(e\right)})\\ I_{l}=\overset{p\times m}{\underset{i=1}{\vee }}I_{il}\;{\hat{y}}_{i}^{\left(v\right)} \end{array}$	The output of the exteroceptive autoassociative network, $\hat{a}$^(*e*)^, drives retrieval in the heteroassociative network. An intermediate valence cell, *l*, can fire (*y*^(*v*)^_*l*_ = 1) only if the dendritic sum of its excitatory inputs exceeds the specified threshold ($\hat{y}$^(*v*)^_*l*_ = 1) and if it does not receive inhibitory inputs from other valence cells that have already fired (*Ī*_*j*_ = 1 or *I*_*j*_ = 0).
R3	$\begin{array}{l} {\hat{a}}_{j}^{\left(i\right)}=H(\sum_{i=1}^{m}w_{ij}^{\left(i\right)}{\tilde{a}}_{i}^{\left(i\right)}-\sum_{i=1}^{m}{\tilde{a}}_{i}^{\left(i\right)})\\ {\tilde{a}}_{k}^{\left(i\right)}=\overset{p\times m}{\underset{i=1}{\vee }}\;w_{ik}^{\left(v-i\right)}y_{i}^{\left(v\right)} \end{array}$	The activity of the intermediate valence cells serves as input to the interoceptive autoassociative network through prewired connections, *w*^(*v* − *i*)^_*ik*_. The initial input is denoted by $\tilde{a}$^(*i*)^ and $\hat{a}$^(*i*)^ specifies the retrieved output, via recurrent connections, *w*^(*i*)^_*ij*_.
**LEARNING**		
L1	$w_{ij}^{\left(e\right)}(t+1)=w_{ij}^{\left(e\right)}(t)\vee (a_{i}^{\left(e\right)}\wedge a_{j}^{\left(e\right)})$	The activity state of *n* cells in the exteroceptive autoassociative network is determined only by afferent inputs from the EC, *a*^(*e*)^ ∈ {0, 1}^*n*^. The recurrent synaptic weight *w*^(*e*)^_*ij*_ between two cells *i* and *j* is clipped at 1 if both cells are active; otherwise, no change occurs. Initially, *w*^(*e*)^_*ij*_(0) = 0.
L2	$w_{ij}^{\left(i\right)}(t+1)=w_{ij}^{\left(i\right)}(t)\vee (a_{i}^{\left(i\right)}\wedge a_{j}^{\left(i\right)})$	*a*^(*i*)^ ∈ {0, 1}^*m*^ specifies interoceptive, valence, activity pattern from the EC and *w*^(*i*)^_*ij*_ specifies synaptic weights of the interoceptive autoassociative network. Initially, *w*^(*i*)^_*ij*_(0) = 0.
L3	$\begin{array}{l} w_{ij}^{\left(e-v\right)}(t+1)=w_{ij}^{\left(e-v\right)}(t)\vee (a_{i}^{\left(e\right)}\wedge h_{j}^{\left(v\right)})\\ h_{j}^{\left(v\right)}=x_{j}^{\left(v\right)}\wedge {\bar{I}}_{j}\\ x^{\left(v\right)}=(m_{1}a^{\left(i\right)},m_{2}a^{\left(i\right)},\ldots ,m_{p}a^{\left(i\right)})\\ y^{\left(v\right)}=(g_{1}^{\left(v\right)},g_{2}^{\left(v\right)},\ldots ,g_{p}^{\left(v\right)}) \end{array}$	The intermediate valence cells are organized into *p* groups of *m* cells. As long as no inhibition is exerted on the primary group, its cells can be activated directly by interoceptive inputs from the EC (*m*_1_ = 1). The interoceptive input, *a*^(*i*)^, on the remaining *p* − 1 groups of valence cells is gated by being multiplied by *m*_*k*_ signals that coincide with the detection of interference (Ξ_*k* − 1_ = 1). The interference condition verifies when the distance (HD) between the retrieval activity, *y*^(*v*)^_*j*_, across the valence cells of a group *k* (*g*^(*v*)^_*k*_) and the interoceptive activity from the EC (*a*^(*v*)^) exceeds a threshold *v*. Initially, *w*^(*e* − *i*)^_*ij*_(0) = 0.
	$\begin{array}{l} m_{1}=1,\;\;m_{k\in [2,p]}=\{\begin{array}{l} 1,\;\;\;\text{if}\;\;\Xi_{k-1}=1\\ 0,\;\;\;\text{otherwise} \end{array}\\ \Xi_{k}=\sum_{i=1}^{m}g_{k_{i}}^{\left(v\right)}>0\;\;\wedge \;\;\text{HD}(g_{k}^{\left(v\right)},a^{\left(v\right)})>v \end{array}$	

The model receives two kinds of sensory inputs, *a*^(*e*)^ and *a*^(*i*)^, reflecting exteroceptive and interoceptive patterns of activity of afferent input from the EC (Figure 1A). Interoceptive patterns may reflect the emotional valence (pleasantness of the events), arousal (the intensity of emotion provoked by the experienced events) and other somatic states (e.g., hunger or satiety) of external stimuli. However, for simplicity, we focus on the valence of the presented stimuli but all computations in the model generalize trivially to other interoceptive features.

Two autoassociative networks are considered in the model. They receive independently exteroceptive and interoceptive input patterns, *a*^(*e*)^ and *a*^(*i*)^, via one-to-one connectivity with EC cells. The first network operates, as it is usually the case, to link exteroceptive features of a given stimulus by strengthening recurrent synaptic weights, *w*^(*e*)^_*ij*_, between co-active cells. The cells in the second autoassociative network serve a similar function for interoceptive features via their recurrent connections, *w*^(*i*)^_*ij*_.

In addition, the model includes a small number of ordered groups of intermediate valence cells that receive stimulus valence from the same interoceptive pathways. As long as no inhibition is exerted on the first group of intermediate valence cells, its cells can be directly activated by interoceptive inputs to the model and are therefore considered as the primary valence cells. Interoceptive inputs on the cells in the other groups, which are termed associated cells, are conditional. This means that interoceptive inputs can not evoke postsynaptic activity within the associated cells unless a concomitant signal *m*_*k*_ emanating from a precedent group of valence cells is applied. The occurrence of *m*_*k*_ signals coincides with the detection of interference at the level of the activity pattern of the corresponding group of valence cells (cf. Section 2.2 and Equation L3 in Table 2).

The cells within the same group of intermediate valence cells are not interconnected but inhibitory connections, *I*_*ij*_, exist between cells belonging to different groups. These connections are not plastic, they are prewired such that an inhibitory connection from cell *i* to cell *j* exists (*I*_*ij*_ = 1) if the two cells belong respectively to different groups, *k* and *l*, and *l* precedes *k*. Thus, each group of associated cells, once activated, exerts a shunting-type inhibitory effect on its preceding groups including the primary group of valence cells, and at most valence cells in one group can be active at a time.

The coupling of exteroceptive sensory information to their emotional valences occurs at the level of heteroassociative links, *w*^(*e* − *v*)^_*ij*_, between the exteroceptive autoassociative network and intermediate valence cells. These latter provide, in turn, direct excitatory input to the interoceptive autoassociative network through prewired connections, *w*^(*v* − *i*)^_*ij*_. Specifically, a connection (*w*^(*v* − *i*)^_*ik*_ = 1) is wired if cells *i* and *k* are sensitive to the same kind of valence; otherwise, *w*^(*v* − *i*)^_*ik*_ = 0.

The standard binary version of the Willshaw associative network (Steinbuch, 1961; Willshaw et al., 1969) is used as a basis to simulate both auto- and heteroassociative memory functions in the model. It is one of the most efficient associative memory networks as long as the patterns to be stored are sparse (Graham and Willshaw, 1997), and is often used for modeling neural circuits across brain regions (Palm, 2013), and widely adopted for estimating the potential storage capacity in the hippocampus (Bennett et al., 1994; Dubreuil et al., 2014).

The Willshaw's heteroassociative network is a feedforward network with two layers of McCulloch-Pitts binary threshold cells. The *n* cells of the input layer are fully connected to *m* cells in the output layer through modifiable binary synapses, *w*_*ij*_ ∈ {0, 1}^*n* × *m*^. The network can store *M* pairs of binary patterns presented at the input and output layers ({(*x*^μ^, *y*^μ^)}, *x*^μ^ ∈ {0, 1}^*n*^, *y*^μ^ ∈ {0, 1}^*m*^, μ = 1 … *M*), by setting synaptic strengths according to a clipped version of Hebbian learning: a single coincidence of presynaptic and postsynaptic activity (*x*^μ^_*i*_ = 1 and *y*^μ^_*j*_ = 1) is enough to change the synaptic weight *w*_*ij*_ from 0 to 1, while further co-activations do not induce further changes.

For identical input and output patterns (*x*^μ^ = *y*^μ^ and *n* = *m*), it is trivial to configure the Willshaw's network to act autoassociatively as a single-layer network with *n* cells connected through recurrent synaptic connections, *w*_*ij*_ ∈ {0, 1}^*n* × *n*^.

When the network is used for recall by presenting a cue pattern $\tilde{x}$ on the input layer, a pattern of output activity can be recalled if dendritic potential ($s_{j}=\sum_{i=1}^{n}w_{ij}{\tilde{x}}_{i}$) exceeds a global firing threshold θ. A well-selected value of the threshold is important for successful recall. Here we use the classical threshold as proposed by Willshaw et al. (1969), that is, the number of active cells in the input ($\theta =\sum_{i=1}^{n}{\tilde{x}}_{i}$). This strategy is simple and biologically plausible as it can be implemented, for example, through feedforward inhibition (Knoblauch et al., 2010). The quality of a recalled pattern can be assessed according to its difference from the originally stored pattern. Here we use Hamming distance as a basis for the assessment of recall quality. Hamming distance between two binary patterns x and y, HD(x, y), is computed as the number of elements that differ between the patterns. For example, if x = (0 1 1 1 0) and y = (1 1 0 1 0) then HD = 2.

### 2.2. Storage and recall

The model's dynamics are largely based on the assumption—shared by many other hippocampal models (Hasselmo et al., 1996; Meeter et al., 2004)—that the hippocampus generates its own novelty signal and uses it as a basis for self-aligned transitions between storage and recall modes. The essential idea is that when novel patterns are presented to the hippocampus an inhibitory effect is exerted on the septum leading to strong increase of acetylcholine (ACh) release from septal cholinergic projections to the hippocampus. The increase in Ach appears to give rise to specific network dynamics that favor response to afferent inputs while decreasing the synaptic transmission at intrinsic modifiable synapses during learning (Hasselmo, 2006).

The model is simulated in discrete time steps, that is, the activity states of cells/synapses at time step *t* determine the next states at time *t* + 1. The mathematical details of the model equations that govern the learning and recall processes are presented in Table 2.

The recall process starts by presenting the exteroceptive autoassociative network with a particular pattern of activity, *a*^(*e*)^, from the EC. The activity states of cells in the autoassociative network are then updated according to the total recurrent excitatory activity they receive. This yields the output pattern, $\hat{a}$^(*e*)^, which corresponds to pattern completion of exteroception. Similarly, the activity, $\hat{a}$^(*e*)^, propagates along heteroassociative links and elicits activation of groups of intermediate valence cells, *y*^(*v*)^. Due to the inhibitory interactions between these groups, at most valence cells in one group can become active. This activation can trigger recall in the interoceptive autoassociative network and a third pattern of activity, $\hat{a}$^(*i*)^, emerges at the output of the network which corresponds to valence prediction (Figure 1).

Just after the completion of the exteroceptive pattern coming from the entorhinal cortex (EC), a novelty-detection process takes place to compare the retrieved pattern, $\hat{a}$^(*e*)^, to the actual pattern in the EC, *a*^(*e*)^. The mismatch/novelty condition occurs when the Hamming distance between the two patterns exceeds a pre-specified threshold (HD^(*e*)^ > *e*). If the EC activity pattern corresponds to the retrieved pattern, valence prediction can be signaled to other brain regions such as the amygdala.

Upon receipt of interoceptive information, a similar process occurs to evaluate prediction error in terms of Hamming distance between actual *a*^(*i*)^ and predicted values $\hat{a}$^(*i*)^. Likewise, novelty occurs when the distance exceeds a pre-specified threshold (HD^(*i*)^ > *v*).

Errors in valence prediction can be related to two separate factors: (1) It may be that the model does not have any prediction for exteroceptive cues (if novel exteroceptive cues) or (2) may stem from overload interference at the level of heteroassociative links between exteroceptive patterns and their associated valences (valence overload). In the first case, it would be sufficient to store the new inputs and their association to be retained and recalled later. Direct learning of new associations in the second case will continue to interfere with the recall of older, valence-related information.

The model deals with valence-overload interference by monitoring activity of intermediate valence cells, *y*^(*v*)^, during recall. If any activity is observed among intermediate valence cells (∑_*i*_ *y*^(*v*)^_*i*_ > 0) in response to exteroceptive cues a matching process takes place to determine whether this activity matches valence-related activity in the EC. A mismatch (HD^(*v*)^ > *v*) signals a potential interference to a successive group of associated valence cells that become able to respond to valence-related inputs from the EC and rapidly silence valence cells that were active in preceding groups.

Both exteroceptive inputs with high novelty (HD^(*e*)^ > *e*) and valence prediction errors (HD^(*i*)^ > *v*) can induce dynamics that favor learning of new inputs, otherwise the model settles in recall mode.

During learning, excitatory intrinsic synaptic transmission is removed and activity in the model is purely driven by afferent inputs from the EC, *a*^(*e*)^ and *a*^(*i*)^. Synaptic weights at the level of auto- and heteroassociative networks are then updated according to Equations (L1–L3) in Table 2.

**Figure 2:**
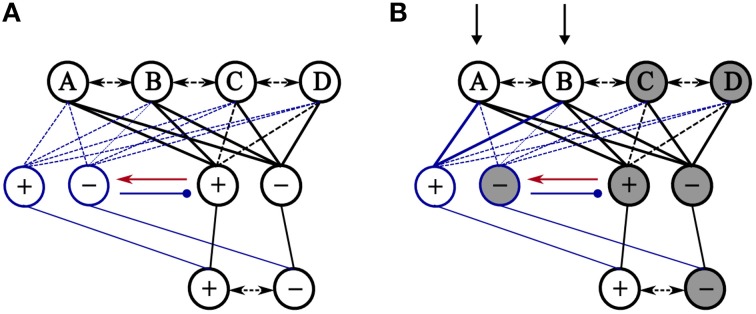
**Illustration of how accumulated learning of emotional stimuli causes valence interference and how the proposed model attempts to solve it**. **(A)** Initially, the model is trained on three emotional patterns (AB+, AC−, BD−) where + indicates positive valence and – denotes negative valence. Thick solid lines between exteroceptive layer and valence cells are used to denote learned connections. The figure shows that the learning of AC and BD patterns linked irrelevantly the components of AB to negative valance. **(B)** Presentation of AB as a retrieval cue leads to the activation of both cells in the primary group of valence cells. After the receipt of the actual valence (+) of the pattern (AB), prediction error at the output of the model causes its dynamics to favor learning, and concurrently the false pattern of activity in the primary group of valence cell triggers a mismatch signal to the next group of associated cells (red). This allows the positive valence-specific cell in that group to fire while cells in the primary group fall silent by inhibitory effects of the associated group of valence cells.

As an example, Figure 2 shows how the model deals with the case of the three emotional patterns (AB+, AC−, BD−) discussed above. Following the encoding of the three patterns, the presentation of AB will activate cells involved in coding positive and negative valence. This pattern of activity triggers a mismatch signal allowing the activation of a new group of associated cells by EC afferent inputs. Then, a new encoding of AB+ proceeds with the new associated cells. A subsequent presentation of AB will activate that associated cells which, in turn, inhibit preceding valence cells allowing an accurate prediction of its associated valence.

### 2.3. Simulation methods

To validate the model outlined above, several simulations were run. The stimulated model contains 300 cells for the exteroceptive autoassociative network, 3 cells for the interoceptive, valence, autoassociative network. The intermediate valence cells are organized into one primary group of 3 cells and 4 groups of 3 associated cells.

The entorhinal inputs are provided to the model as two independent patterns of activity. The exteroceptive inputs are modeled as a randomly constructed pattern of activity across 300 binary cells. The number of active cells was held fixed at 8 (≃ *log*_2_(300), a maximal level of density allowing for a successful recall in the Willshaw model) except where a partial cue is used to elicit recall of previously stored information. To reflect the emotional valence associated with exteroceptive patterns, 3 binary cells were used to differentiate positive, negative and neutral valence states. One of these cells switches to its active state according to whether a pleasant (100), unpleasant (010), or neutral (001) stimulus is present. The novelty-detection thresholds, *e* and *v*, are set to zero, except for simulations described in Section 3.3.

The pattern of activity being recalled at the output of the model corresponds to both pattern completion of exteroceptive cues and valence prediction. Therefore, two kinds of recall errors are considered when evaluating simulation results. One reflects the mismatch between the stored and retrieved activation for exteroceptive patterns. The other reflects the difference between the predicted and correct valence. In both cases, errors are defined in terms of Hamming distance. No error implies a Hamming distance equal to zero, while error means a Hamming distance greater than zero.

To ensure that the effects observed in the model stem from valence overload interference at the level of heteroassociative links, the model is tested under the assumption of perfect storage and recall within the autoassociative networks (pattern overload interference can not occur at the level of autoassociative memories). That is, the memory load is kept low to ensure an error-free recall of stored exteroceptive patterns from full cues.

We start with a generic setup to examine the behavior of the model under a number of different conditions (such as the number of stored patterns, and recall under full-cue vs. partial-cue conditions). Our model is contrasted to a standard autoassociative model, i.e., a single autoassociative network in which both extero- and interoceptive inputs are considered as two parts of a single pattern, as well as to a simple heteroassociative model which can be thought of as a reduced version of the proposed model without the groups of associated cells.

**Figure 3:**
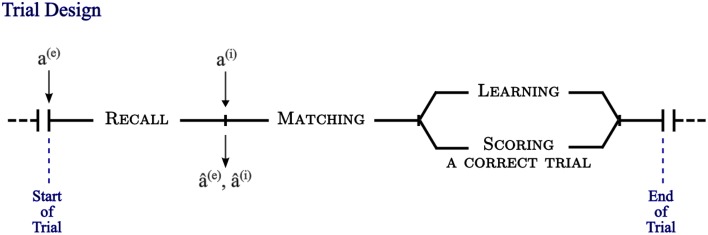
**A trial, as experimentally designed, is composed of three phases**. The first phase is a recall process triggered by the presentation of an exteroceptive pattern, *a*^(*e*)^, as a retrieval cue at the input of the model. At the end of this phase, two patterns of activity emerge at the output of the model: $\hat{a}$^(*e*)^ corresponding to pattern completion of exteroception, and $\hat{a}$^(*i*)^ corresponding to valence prediction. The second phase begins with the delivery of the original valence pattern, *a*^(*i*)^, and two matching processes take place to compute pattern completion and valence prediction errors. In the third phase a learning process occurs in case of error/mismatch, otherwise a correct trial is scored and the next trial begins.

The simulation experiments are organized in terms of one or more blocks of trials (Figure 3). A block of n trials consists of one pass through n extero-interoceptive patterns that are randomly ordered in each block. A training trial begins with the presentation of an exteroceptive pattern *a*^(*e*)^ as a retrieval cue to drive the recall process in the model. At the end of this process, two patterns of activity emerge at the output of the model: $\hat{a}$^(*e*)^ which corresponds to pattern completion of exteroception, and $\hat{a}$^(*i*)^ which corresponds to valence prediction. Immediately after that, the original valence pattern, *a*^(*i*)^, is delivered in order to evaluate the performance of the model and determine whether it is necessary to shift the model into a learning mode to store the externally presented patterns. Following the end of a given experiment, errors are recorded over a block of testing trials in which learning is disabled.

Next, we build upon a two-phase learning paradigm established by Levy-Gigi et al. (2011) to investigate reversal learning in patients with amnesic mild cognitive impairment (aMCI) which may arise from hippocampal pathology. The first phase is a discrimination learning task in which participants were presented with four different cards containing a shape representing a cue and a background representing a context. Two of the cards are associated with positive outcome (winning points) whereas the others are associated with negative outcome (loosing points). Acquisition was followed by a reversal phase where an additional set of 8 new cards was used with two possibilities: A new card can share the same cue as an original card but appears in a new context or a new cue can be presented in the original context. The new cards were associated with the opposite outcome relative to the discrimination learning phase. The reversal phase was a series of retention and reversal trials. On retention, the original cards were presented with the same outcome, whereas cards with a new context or a new cue were presented on reversal trials. Participants have to learn to reverse their choice without a change in the relevant feature for a cue or a context. To simulate this task, we generated three groups of 4 exteroceptive patterns each. One of the 8 active cells is used to encode the presence of a cue and the others to encode the presence of a context. No overlap is allowed between cells encoding for different cues or contexts. That is, 8 different cells are used to encode 8 different cues and 56 cells are used to encode 8 different contexts. Positive and negative valences are associated with the 12 patterns the same way as described above.

All simulation results reported in the next section are averaged over 20 simulation runs with different random patterns, and are presented with a confidence interval of 95%.

## 3. Results

### 3.1. Predictive recall using full exteroceptive cues

In general, associative networks show a degradation in recall quality as the number of stored patterns increases. Here we investigate the effect of the number of stored patterns on valence prediction when no degradation in recall occurs at the level of exteroceptive patterns. Our model, as well as standard autoassociative and heteroassociative models, were trained on blocks of N patterns that were randomly assigned to a positive, negative or neutral valence.

**Figure 4:**
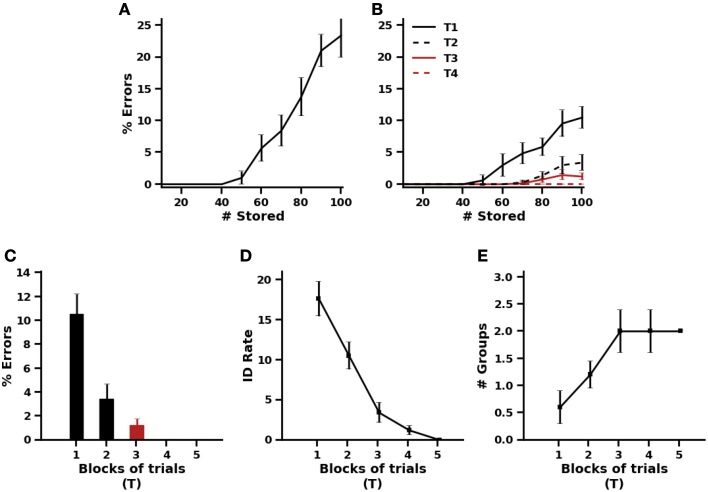
**Effect of the number of stored patterns on valence prediction**. **(A)** Percentage of prediction errors of the standard autoassociative and heteroassociative models. **(B)** Percentage of prediction errors of the proposed model after one to four blocks of training trials. **(C,D,E)** Details on the behavior of the proposed model trained on blocks of 100 patterns. **(C)** Prediction errors across 5 blocks of trials. **(D)** Rates of interference detection over each block of trials. **(E)** Number of groups of associated cells needed to resolve interference across the 5 blocks of trials.

No differences were observed between standard autoassociative and heteroassociative models. Taken together in Figure 4A, results show that for a set of 10, 20, and up to 40 patterns the models make correct prediction. As the number of stored patterns increases to 50 or more, the models continue to reliably retrieve stored exteroceptive patterns but prediction errors begin to occur more frequently, reaching about 25 of a total of 100 patterns. The explanation lies in the fact that the number of exteroceptive features far outnumbers that for valence, then interoceptive features are much more willing to be shared between stored patterns. This suggests that additional irrelevant connections tend to be strengthened as long as novel patterns are being stored. On the other hand, our model detects the effect of such irrelevant connections and attempts to prohibit interference from occurring by promoting the recruitment of a successive group of associated cells. Figure 4B shows how prediction errors are significantly reduced after the first presentation of patterns and reach zero after few additional presentations. In particular, at *N* = 100 stored patterns, Figure 4C demonstrates that a gradual reduction in prediction errors is observed across 4 blocks of training trials due to the detection of a number of potential problems of interference over each block of trial (Figure 4D). A maximum of two groups of associated cells is needed to reduce prediction errors to zero (Figure 4E).

### 3.2. Predictive recall using partial exteroceptive cues

Despite having observed in the simulations presented above that recall proceeds in much the same way in standard autoassociative and heteroassociative models, a functional difference is to be expected when valence prediction is done on the basis of partial exteroceptive cues. In a standard autoassociative model, pattern completion and valence prediction are performed concurrently while a heteroassociative model supports successive recall processes in such a way that valence prediction could be performed from a full representation of exteroceptive patterns resulting from pattern completion under partial cue condition. Based on this, one might predict that autoassociative models would be more prone to prediction errors than heteroassociative models.

**Figure 5:**
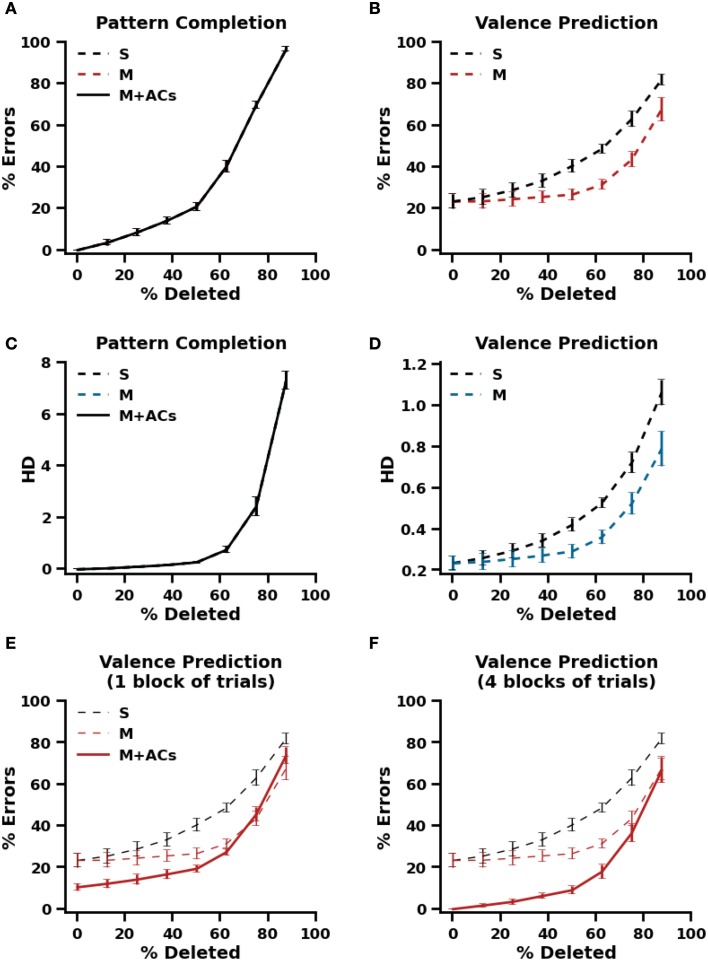
**Recall performance using partial cues**. **(A)** Pattern completion performance, defined as the percentage of retrieved patterns that differ from the stored patterns by one element at least, in a standard autoassociative model (S) and a heteroassociative model (M). **(B)** Valence prediction in the standard autoassociative model (S) and the heteroassociative model (M). **(C,D)** Pattern completion and Valence prediction performances, defined in terms of Hamming distance between the stored and retrieved patterns in the standard autoassociative model (S) and the heteroassociative model (M). **(E,F)** Valence prediction in the proposed model (M+ACs, with ACs stands for associated cells) after one block of training trials **(E)** and after 4 blocks of training trials **(F)**.

To test this prediction, standard autoassociative and heteroassociative models were trained on a set of 100 patterns that were randomly assigned to a positive, negative or neutral valence. After training, distinct input patterns were generated from each of the trained exteroceptive patterns, such that a fixed number of active cells become quiescent (set to 0). This number was increased from 1 up to 7 with subsequent testing trials. The partial patterns were presented to the models and recall performance was assessed (Figure 5).

Once again, both autoassociative and heteroassociative models produced the same performance on pattern completion of exteroceptive cues (Figures 5A,C). Both show a gradual decline in pattern completion as the number of active cells decreases but, as shown in Figure 5C, pattern completion is almost perfect (HD ≤ 1) for 1 to 4 cells being silenced or deleted in the originally trained patterns. This appears to provide a clear support for the heteroassociative model to maintain its performance on valence prediction as it was the case without deletion (Figures 5B,D). These results also agree with the prediction discussed above, that exteroceptive pattern completion being performed prior to valence prediction in the heteroassociative model makes it more accurate than the autoassociative model, even when only a small number of originally active cells is presented to the model. Similar functional improvement is observed with the proposed model which is inherently a heteroassociative model (Figures 5E,F). However, the improvement is significantly larger in the proposed model because its learning is better even after one presentation of training patterns.

### 3.3. Phasing storage and recall

Since the number of exteroceptive features is grossly disproportionate to that of interoceptive features, our model makes use of two novelty signals to mediate transitions between storage and retrieval modes. These signals are generated separately at the level of exteroceptive patterns and their associated valences. In this way the model can grant more tolerance of exteroceptive errors than interoceptive errors while being able to distinguish between different conditions where input patterns should be treated as novel.

**Figure 6:**
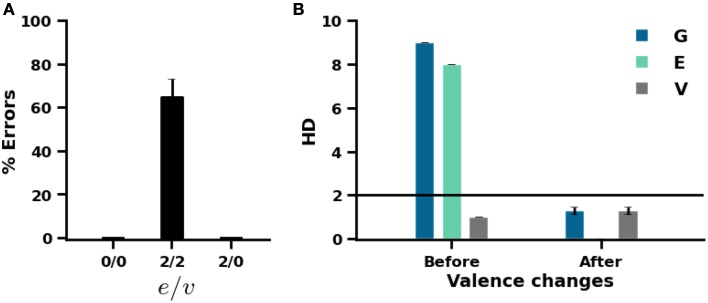
**Effect of novelty thresholds on the performance of the model. (A)** Errors in valence prediction for different values of exteroceptive (*e*) and interoceptive (*v*) thresholds. **(B)** Hamming distance between input and output patterns before and after valence changes. Hamming distance is calculated in overall (G) and separately for exteroception (E) and valence (V).

To illustrate this behavior, the proposed model was initially trained on a set of 10 patterns that were associated with neutral valence. Whatever the values of novelty thresholds *e* and *v* (*e*/*v* = 0/0, 2/2, 2/0), the model identified each of these patterns as novel because the difference at the level of exteroceptive patterns is much higher (see Figure 6B). Therefore, learning has occurred. The valence of these 10 patterns is then randomly repeated or changed to positive or negative values. The model was retrained on these patterns to allow it to adapt to valence changes. Subsequently, the model is tested for valence prediction. As shown in Figure 6, when both thresholds are set to 2, the model produces prediction errors on about two-thirds of the trained patterns. The model was not able to detect changes in valence because exteroceptive patterns were recognized by the model (HD = 0, see Figure 6B), so no learning has occurred. By contrast, when valence threshold was set to 0 whatever the value of exteroceptive threshold, 0 or 2, the model offers an errorless performance due to the successful detection of valence changes.

### 3.4. Cue-context reversal learning

Similarly to the task of Levy-Gigi et al. (2011), the proposed model, as well as the standard autoassociative and heteroassociative models, are applied to initially acquire a discrimination between a set of positively or negatively valenced patterns, and then to switch its choice following a reversal at the level of cues or contexts in the original patterns. The experimental design of the task is summarized in Table 3.

**Table 3:** **The experimental design of the task of Levy-Gigi et al. (2011)**.

**Training patterns**			**Task**	
**Group 1 (Original)**	**Group 2 (Cue reversal)**	**Group 3 (Context reversal)**	**Phase 1 (Acquisition)**	**Phase 2 (Retention and Reversal)**
A1+	E1−	A5−	Group1	Group1
B2+	F2−	B6−		Group2
C3−	G3+	C7+		Group3
D4−	H4+	D8+		

*A–H refer to eight cue shapes, 1–8, eight contexts, + and − indicate respectively positive and negative valences*.

**Figure 7:**
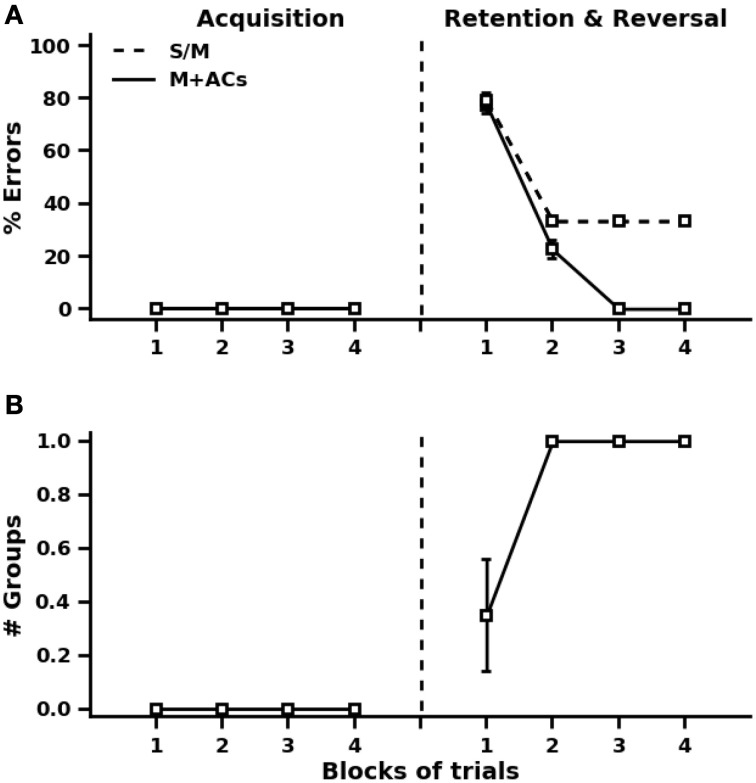
**Simulation results of the reversal learning task of Levy-Gigi et al. (2011). (A)** Performance on valence prediction before and after cue and context reversal learning using the standard autoassociative and heteroassociative models (S/M) and the proposed model (M+ACs, with ACs stands for associated cells). **(B)** Number of groups of associated cells needed by the proposed model during acquisition and reversal phases.

In the first phase of acquisition, the models are repeatedly presented with the training patterns in the first group and valence prediction is evaluated over four blocks of four trials. As in previous simulations, the order of training trials is randomized within each block. There was no difference in the performance of the three models during the acquisition phase (Figure 7A). All the three models make correct valence prediction after a single exposure to the training patterns.

In the reversal phase, the models are exposed to new training patterns from the second and third groups, in addition to the old ones. The 12 training patterns are presented repeatedly four times in random order across four blocks of trials. In the initial stage after this reversal, valence prediction errors drastically increase for all the three models (Figure 7A). This is quite expected because more than 66% of the training patterns are new. It is also obvious from the results of Figure 7A that valence prediction errors are made for old patterns as well. This reflects the fact that heteroassociative connections are irrelevantly strengthened between the original patterns and valences of new patterns. The proposed model detects all interference problems on the first two blocks of training trials after reversal and engages accordingly one group of valence-associated cells to deal with interference effects (Figure 7B). The standard autoassociative and heteroassociative models, on the other hand, show impaired valence prediction performance on the old patterns after learning the new ones. This impairment persists over repeated trials since old exteroceptive patterns become associated with both positive and negative valence values.

## 4. Discussion

### 4.1. Autoassociative and heteroassociative hippocampal networks

The model proposed in this paper is developed to elucidate neural mechanisms that might underlie the rapid, one-trial learning of extero-interoceptive associations in the hippocampus. The binding of exteroceptive features is accomplished, as it is usually the case, through a separate autoassociative network, subserving pattern completion of exteroceptive patterns. Along with this first autoassociative memory, a second one is employed to serve a similar function for valence features. The two autoassociative networks are interconnected through heteroassociative links, allowing flexible interactions between the exteroceptive stimulus patterns and their associated emotional valences.

By evidence of associative synaptic plasticity at most of the hippocampal synapses (Andersen et al., 2007), auto- and heteroassociative networks have already been validated as prototypical models of the mnemonic function of the hippocampus. While autoassociative learning has long been ascribed to CA3 because of its recurrent connections (Marr, 1971), heteroassociative learning has been reported to occur at the Schaffer collaterals projecting from CA3 to CA1 (Miyata et al., 2013), at CA3 backprojections on hilar mossy cells and DG granule cells (Lisman and Otmakhova, 2001). Recently, the functional homogeneity of CA3 has been called into question (de Almeida et al., 2007; Witter, 2007; Hunsaker et al., 2008; Thompson et al., 2008; Bush et al., 2010). The CA3 subregions (CA3a, b, and c) have been reported to exhibit distinct anatomical and neurophysiological characteristics. In particular, Samura et al. (2008) have proposed, based on the location dependency of the recurrent collaterals of CA3, that both CA3a and CA3b act as autoassociative memories while CA3c acts as heteroassociative memory.

It has been argued that heteroassociative plasticity may underlie the learning of memory sequences in the hippocampus (Lisman and Otmakhova, 2001; Samura et al., 2008). It is also plausible that the heteroassociative properties of hippocampal synapses could serve, as suggested by our model, to facilitate binding between different kinds of information that may be mediated by different regions and circuitry in the hippocampus. Some aspects in favor of this possibility will be discussed in Section 4.2 below.

As opposite to the purely autoassociative way of binding proposed by previous models (e.g., O'Reilly and Rudy, 2001; Rolls, 2010), our model proposes that pattern completion of exteroceptive patterns occurs prior to the prediction of their emotional valences. This allows, as demonstrated by the simulations in Figure 5, for more accurate predictions under partial-cue recall conditions. Note that the validity of this principle is evident for extero-interoceptive associations formed outside the hippocampus. Projections from the hippocampus, including those to the amygdala (Pitkänen et al., 2000), arise mainly from CA1, that is, after completion of exteroceptive patterns in CA3. This may be the reason why the effect of partial-cue removal on valence prediction has not received attention in experimental studies. While this is accepted in principle, such studies would be extremely useful to determine what type of binding is used to integrate exteroceptive and interoceptive information within the hippocampus. A good argument in favor of the heteroassociative connectivity can be obtained if the accuracy on valence prediction does not differ significantly as the number of available cues decreases (up to 50% according to our simulation results).

### 4.2. How does interoceptive information reach the hippocampus?

It is known that sensory information from most unimodal and polymodal associative cortical areas arrives at the superficial layers of the EC and passes to the hippocampus via the perforant path (de Curtis and Paré, 2004). The axons of the perforant path arise mainly in layers II and III of the EC; layer II neurons project directly to the DG and CA3, while layer III neurons project to CA1 hippocampal region (Andersen et al., 2007). In primates, spatial information is conveyed to the superficial layers of the EC by the parahippocampal cortex (PHC) whereas non-spatial information is conveyed by the perirhinal cortex (PRC). According to this view, PRC which receives important projections from the insular cortex (Craig, 2009) might also convey interoceptive information to the hippocampus (Figure 1). Notably, the transfer of sensory inputs from PRC to EC is subject to long-range feedforward inhibition (de Curtis and Paré, 2004; Apergis-Schoute et al., 2007), that has been shown to be counteracted by BLA glutamatergic projections to PRC and EC (Paz and Paré, 2013). Such effect was observed, for example, in the case of the experiment reported by Paz et al. (2006) when a reward is unexpectedly delivered, but attenuated with learning when rewards occur as predicted. This suggests that BLA activity may facilitate the transmission of emotional information under certain conditions to the hippocampus (de Curtis and Paré, 2004).

In addition, unlike exteroceptive information, alternate pathways may provide a route for interoceptive features to reach the hippocampus (Andersen et al., 2007). Direct projections from the insular and orbitofrontal cortices terminate in the deep layers of the EC but they may contribute to the perforant path through intrinsic deep to superficial projections in the entorhinal cortex. The amygdala, which may relay valence related information, exclusively targets cells in layer III of the EC (Canto et al., 2008). Both CA3 and CA1, but not the DG, receive direct projection from BLA. The septum, which interacts closely with limbic structures, is another potential source of interoceptive information that may reach all subdivisions of the hippocampal formation via fimbria-fornix pathway. This pattern of neuroanatomical connectivity argues for a relative segregation between the processing of exteroceptive and interoceptive information in the hippocampus. It is also possible that distinct types of extero-interoceptive associations are mediated by different heteroassociative networks in the hippocampus.

The anatomical and neurophysiological characteristics of CA3 make it attractive as a candidate for the locus where one-trial associations might be formed between diverse exteroceptive and interoceptive inputs. The architecture of the model presented in this paper represents a minor departure from the purely autoassociative models of CA3—since only a small fraction of cells encoding valence are isolated from other cells binding exteroceptive features in a homogeneous autoassociative network. We assume that CA3a, where recurrent connections are most prevalent, is the best fitted to the exteroceptive autoassociative network in the model. But it is harder to predict where the interoceptive autoassociative network might be located as only very little is known about the distribution of valence signals in the hippocampus. However, on the basis of the results of Nakamura et al. (2013) about the presence of a proximal CA3-distal CA1 network involved primarily in processing nonspatial information, it is more likely that interoceptive information is encoded in CA3b,c and/or CA1 of the ventral hippocampus.

Generally, CA3 has been identified to be more involved than CA1 in the rapid encoding of a wide range of novel associations afforded by the hippocampus (Nakashiba et al., 2008; Kesner, 2013). Selective blockade of N-methyl-d-aspartate (NMDA) receptors at CA3-CA3 synapses is associated with impairments on single-trial learning tasks (Lee and Kesner, 2002), including spatial tasks (Nakazawa et al., 2003), paired-associate learning paradigms (Day et al., 2003), and fear memories (Zhang et al., 2001; Quinn et al., 2005). Thus, it is likely that CA3 NMDA receptor-mediated plasticity mechanisms generally contribute to binding distinct aspects of one-trial experiences including their interoceptive features.

Together, these data support our hypothesis that specific subregions of CA3 and CA1, but not the DG, may constitute specific processing nodes of interoceptive information within the hippocampus. Under this view, we have suggested that the hippocampus may recruit different mechanisms by which decorrelated representations can be established for exteroceptive patterns and their associations to interoceptive patterns. This last point is the focus of the next section.

### 4.3. Single-trial learning and valence-overload interference

In this paper, we have argued for a distinction between two possible mechanisms by which the hippocampus may reduce interference: (1) DG projections to CA3, as widely believed, are necessary to enforce a new sufficiently separated representation onto CA3 pyramidal cells, preventing (pattern-overload) interference between the representations of exteroceptive patterns stored autoassociatively in CA3, but (2) interference between valences (valence-overload) is controlled by local circuit interactions within CA3 (or probably CA1, cf. Section 4.2). The role of DG in pattern separation has been extensively characterized in previous studies (Leutgeb et al., 2007; Myers and Scharfman, 2009), which prompted us to concentrate more on how to deal quickly with valence-overload interference, especially under conditions involving one-time experiences.

The most obvious way would be to strengthen correct associations between exteroceptive patterns and their emotional valences and weaken incorrect associations. However, this runs the risk of destroying previously acquired associations because of the distributed, albeit fairly sparse, nature of the representations of exteroceptive patterns. The model proposed here provides an alternative mechanism that does not involve forgetting, but exerts rather inhibitory effects to resolve interference from prior learned associations. In particular, the model assumes a reciprocal interplay between groups of valence-associated cells, mediated by excitatory and inhibitory connections such that only cells in one group can fire. When the prediction produced at retrieval does not match the targeted valence, learning is directed to the next group of valence cells that would later inhibit the erroneous response of the former group during recall.

In support of this idea of functionally prewired groups of valence cells are lineage studies demonstrating that clonally related pyramidal cells are organized into discrete clusters in the stratum pyramidale of the hippocampus (Grove and Tole, 1999). More insights have recently been gained into the developmental prewiring of hippocampal circuits (Xu et al., 2014). In particular, as it is the case in our model, it has been found that sister cells do not preferentially form direct contacts with each other, but share common inhibitory inputs from nearby fast-spiking interneurons. Selective connectivity like this suggests that precise inhibitory microcircuits may account for specific activity-dependent processes in the hippocampus.

By the same token, the diversity of excitatory and inhibitory cells in CA hippocampal regions (Bilkey and Schwartzkroin, 1990; Scharfman, 1993) may provide a potential substrate for the detection of interference and the processing of emotional information in the hippocampus. In CA3c, pyramidal cells display some morphological and electrophysiological characteristics different from pyramidal cells in other subregions of CA3. Importantly, it has been found that these cells are not as susceptible to be activated as other CA3 pyramidal cells by stimulation of dentate mossy fibers in hippocampal slices (Scharfman, 1993). Differences in firing patterns exhibited by different classes of pyramidal cells have been observed experimentally in CA3b (Hemond et al., 2008). It is conceivable, therefore, that special mechanisms may be in place to control CA3 neural responsiveness to external inputs under specific physiological conditions.

One may also hypothesize a potential role for neuromodulators in the detection of interference and the processing of emotional information in the hippocampus. The projection pattern of all major neuromodulator systems differs along the septotemporal axis of the hippocampus, with a higher density in the ventral hippocampus (Strange et al., 2014). The magnocellular basal forebrain cholinergic system sends efferents that release ACh in all hippocampal regions. As mentioned previously, and in line with our model, ACh has been implicated in the regulation of oscillatory dynamics in the hippocampus (Hasselmo et al., 1996; Hasselmo, 2006). Increased release in ACh has been proposed to enhance the dynamics of memory encoding by inhibiting synaptic transmission and facilitating long-term potentiation (LTP) at specific connections, which may reduce interference from previously encoded memories during the learning of incoming inputs. Moreover, levels of hippocampal ACh release have been demonstrated to control amygdala function in fear conditioning allowing for adaptive selection of the best predictive stimulus (simple tone vs. context in Calandreau et al., 2006). The influence of other neuromodulators, such as dopamine and norepinephrine, is much less well understood but might be modality-specific, allowing for differential encoding of nonspatial information in the hippocampus (Ito and Schuman, 2012).

It is evident that a great deal remains to understand neural processing of valence-related information in the hippocampus. Yet, as a step in that direction, a testable prediction made by our model is that dentate pattern separation may be needed to decorrelate highly similar exteroceptive patterns but interference between valences may be detected and treated in CA hippocampal regions relying on local inhibitory microcircuits. However, valence-overload interference may not manifest until the number of learned extero-interoceptive associations increases considerably (cf. Section 3.1, Figure 4), due to high decorrelation between hippocampal representations of exteroceptive patterns.

### 4.4. Role of prediction-error signals in the hippocampus

In our model, we assume the existence of two novelty signals, instead of only one, for the ability to identify situations in which experiences are not novel but the prediction of their associated valences is incorrect due to a faulty learning or a change in the previously learned valences (cf. Sections 3.3, 3.4, Figure 6). This view is in accordance with electrophysiological recording studies showing that hippocampal cells differentiate between correct and error responses immediately after the behavioral response (Wirth et al., 2009), and with significantly stronger signals in CA1 (Lee et al., 2012). However, the influence of prediction errors on hippocampal network dynamics, as suggested in our model, has not been examined in the literature. Thus, the relationship between prediction errors and ACh level changes in the hippocampus needs to be further tested empirically in the future. This may also enhance our understanding of the interactions between the hippocampus and other MTL structures, especially the amygdala, as many studies (McIntyre et al., 2002; Calandreau et al., 2006) suggest alterations in hippocampal ACh release as a potential determinant of the relative contributions of MTL structures in learning and memory.

In conclusion, the model proposed in this paper provides an integrated view of how the hippocampus may integrate exteroceptive and interoceptive information and raises the possibility that local inhibitory microcircuits may be recruited to deal with valence-overload interference under the constraint of single-trial learning of episodic memories. Extending our model to include a dentate gyrus network mediating rapid separation of the neural representations of exteroceptive patterns will allow for more complex tasks to be simulated by the model. A further question that remains for future work is to explore how the model will actually operate, synergistically and/or competitively, with other models supporting incremental learning of extero-interoceptive associations. Such a study, we believe, would help explain many puzzling observations relative to hippocampal-dependent behaviors. These include, for example, animal data showing that single- but not multiple-trial contextual fear conditioning is impaired by hippocampal lesions (Wiltgen et al., 2006), while damage to the hippocampus after conditioning causes retrograde amnesia for contextual fear memories regardless of training conditions (Sutherland et al., 2008; Broadbent and Clark, 2013).

### Conflict of interest statement

The authors declare that the research was conducted in the absence of any commercial or financial relationships that could be construed as a potential conflict of interest.
